# Dressing Disks

**Published:** 1883-07

**Authors:** 


### Dressing Disks.

Within the last two or three years the corundum paper disk has been used to some extent by dentists for dressing proximate fillings and the adjacent portions of the teeth. Corundum paper is obtainable of several grades, from that which is sufficiently coarse for rapid cutting, to that which is fine enough for finishing fillings. These can be procured already prepared at the dental depots, but the desired grades cannot always be found there. It is an easy matter for every dentist to make them, and just the kind he may need. Procure a good quality of corundum paper of the hardware dealer. Upon the paper side of the sheet, put one or two good coats of shellac varnish; after this is thoroughly dry, place from three to six sheets one upon another, then with a good gun-wad punch and a hammer, cut out the disks. In this way hundreds, yes, thousands can be made in a short time. For centering, a pin may be put into the punch, or they may be centered after they are cut, by placing from 25 to 50 evenly together, finding the center and driving a sharp instrument through the pack.

They are to be used upon the screw-head mandril. They may be used either singly when the space is small, or a filling to be dressed in but one tooth, or if there are two proximate fillings to be dressed, two disks may be put on the same mandril, with the varnished sides together, they then cut in opposite directions, and will work together perfectly. Care should be taken that the screw of the mandril is drawn tightly.

For using these, as well as the diamond disks, the suspension engine is far preferable to any other, indeed, for the diamond disk no other engine is efficient or safe. The Bonwill engine, however, is next to the suspension in practicability, and would be very good, were it constructed somewhat more carefully, especially with reference to its ease of running and security of the band.

The great difficulty, in the general use of disks of all sorts, is the want of a proper engine.

Cannot the manufacturers give us something that will meet this want? With the present facilities, if the best thing is to be done, two engines are required.

